# A systematic review of mechanistic models used to study avian influenza virus transmission and control

**DOI:** 10.1186/s13567-023-01219-0

**Published:** 2023-10-18

**Authors:** Sébastien Lambert, Billy Bauzile, Amélie Mugnier, Benoit Durand, Timothée Vergne, Mathilde C. Paul

**Affiliations:** 1 IHAP, Université de Toulouse, INRAE, ENVT, Toulouse, France; 2 NeoCare, Université de Toulouse, ENVT, Toulouse, France; 3 Epidemiology Unit, Laboratory for Animal Health, French Agency for Food, Environment and Occupational Health and Safety (ANSES), Paris-Est University, Maisons-Alfort, France

**Keywords:** Avian influenza, modeling, systematic review, control strategies, disease transmission, poultry, simulations, dynamics

## Abstract

The global spread of avian influenza A viruses in domestic birds is causing increasing socioeconomic devastation. Various mechanistic models have been developed to better understand avian influenza transmission and evaluate the effectiveness of control measures in mitigating the socioeconomic losses caused by these viruses. However, the results of models of avian influenza transmission and control have not yet been subject to a comprehensive review. Such a review could help inform policy makers and guide future modeling work. To help fill this gap, we conducted a systematic review of the mechanistic models that have been applied to field outbreaks. Our three objectives were to: (1) describe the type of models and their epidemiological context, (2) list estimates of commonly used parameters of low pathogenicity and highly pathogenic avian influenza transmission, and (3) review the characteristics of avian influenza transmission and the efficacy of control strategies according to the mechanistic models. We reviewed a total of 46 articles. Of these, 26 articles estimated parameters by fitting the model to data, one evaluated the effectiveness of control strategies, and 19 did both. Values of the between-individual reproduction number ranged widely: from 2.18 to 86 for highly pathogenic avian influenza viruses, and from 4.7 to 45.9 for low pathogenicity avian influenza viruses, depending on epidemiological settings, virus subtypes and host species. Other parameters, such as the durations of the latent and infectious periods, were often taken from the literature, limiting the models’ potential insights. Concerning control strategies, many models evaluated culling (*n* = 15), while vaccination received less attention (*n* = 6). According to the articles reviewed, optimal control strategies varied between virus subtypes and local conditions, and depended on the overall objective of the intervention. For instance, vaccination was optimal when the objective was to limit the overall number of culled flocks. In contrast, pre-emptive culling was preferred for reducing the size and duration of an epidemic. Early implementation consistently improved the overall efficacy of interventions, highlighting the need for effective surveillance and epidemic preparedness.

**Supplementary Information:**

The online version contains supplementary material available at 10.1186/s13567-023-01219-0.

## Introduction

Avian influenza viruses (AIVs) pose a continuous threat to domestic poultry, wildlife, and humans. The diversity of two envelope glycoproteins, hemagglutinin and neuraminidase, are used to divide AIVs into various subtypes (named HxNy) with different characteristics and pathogenicity. Waterfowl, especially Anseriformes (ducks, geese and swans) and Charadriiformes (gulls, terns and sandpipers), are considered to be the natural reservoir of all AIV subtypes [1]. When transmitted to poultry, the H5 and H7 AIV subtypes usually cause mild clinical symptoms, and are designated as low pathogenicity avian influenza (LPAI). However, H5 and H7 AIVs can mutate into highly pathogenic avian influenza (HPAI), causing severe morbidity and mortality in poultry. This transition from LPAI to HPAI usually happens in domestic poultry, either shortly after the first introduction of the virus, or after months or even years of undetected circulation [2]. On some occasions, this phenomenon has caused large poultry outbreaks, such as the HPAI H5N2 epidemic in 1983 in the United States, the HPAI H7N1 epidemic in Italy in 1999–2000, the HPAI H7N7 epidemic in 2003 in the Netherlands and the HPAI H7N3 epidemic in 2004 in Canada [3]. Both LPAI and HPAI viruses can be zoonotic, causing a range of symptoms in humans, including severe respiratory distress and death. One notable example of zoonotic AIV infection was the transmission of LPAI and HPAI H7N9 viruses from poultry to humans in China between 2013 and 2017, causing more than 1,500 human cases and over 600 deaths [4].

The emergence of the A/Goose/Guangdong/1/1996 (Gs/Gd) H5N1 virus lineage in China in 1996 [3] has been associated with a shift in HPAI virus transmission. This Gs/Gd lineage has shown a propensity for transmission among wild migratory birds, leading to an unprecedented global spread. Moreover, numerous genome reassortments with other circulating AIVs has led to the diversification of the Gs/Gd lineage into several clades and subclades [5]. From Southeast Asia, HPAI H5N1 viruses first spread to Europe, the Middle-East and Africa in 2005 and 2006. These viruses became endemic in poultry in many countries in Asia and Africa, further promoting virus evolution and diversification.

Beginning in 2014, H5Nx viruses clade 2.3.4.4 became predominant and spread throughout Asia, Europe, Africa, and, for the first time, America. HPAI H5N8 and H5N6 predominated from 2014 to 2021 [5], and were replaced again by HPAI H5N1 starting in 2021 [6]. These viruses caused many outbreaks in domestic poultry; in Europe alone, 2642 H5N8 and 2740 H5N1 HPAI outbreaks have been reported from October 1, 2014 to September 30, 2023 [7]. These viruses even appear to have persisted in wild birds in Europe throughout the summers of 2021 and 2022, suggesting a fundamental shift toward a potential endemic circulation [8, 9]. In addition to devastating losses in poultry, HPAI H5Nx viruses recently caused mass mortality events in wild birds worldwide, raising serious concerns for conservation [10–12].

Control measures against HPAI epidemics in poultry include culling infected birds, pre-emptive culling around infected flocks, movement bans, and screening of at-risk contacts [13]. Poultry vaccination also has been employed in several Asian and African countries [14, 15]. However, this strategy faces several challenges, including difficulties in selecting vaccine strains, monitoring influenza evolution, differentiating vaccinated from infected birds, and maintaining appropriate vaccination coverage [15]. For all of these reasons, poultry vaccination is currently prohibited in the United States (US) [16], and was authorized only recently in the European Union (EU) [17]. Despite significant efforts to limit avian influenza incursions and spread in the poultry sector, the global spread of HPAI viruses, causing devastating epidemics, highlights the need to design and implement adequate prevention and control strategies [18].

Mechanistic models, which describe transmission dynamics by using mathematical expressions, could help in this effort. Models can help evaluate the impact of existing and alternative disease control strategies. They have been used for various animal diseases, including foot-and-mouth disease [19], African swine fever [20, 21], and bluetongue [22]. While numerous mechanistic models have been used to analyze AIV epidemics [23], their results concerning surveillance and control, which could be useful for decision-making, have yet to be subject to a comprehensive review. Reviews conducted so far on avian influenza have focused on virological and clinical aspects [24, 25] or on risk factors [26–28], including transmission routes [29, 30]. Two recent studies reviewed transmission parameter values based on experimental studies [31] and on both experimental and field studies [32], but to the best of our knowledge no comprehensive analysis of mechanistic models has been undertaken. To fill this gap, we conducted a systematic review of mechanistic models applied to avian influenza in poultry to (i) describe the mechanistic models used and their epidemiological context, (ii) list AIV transmission parameters, and (iii) provide insights on avian influenza transmission and the impact of control measures. In regard to our second objective, synthesizing AIV transmission parameters is difficult because parameter values often vary depending on the epidemiological context (e.g., virus subtype, host species, production system). However, listing parameter estimates may be useful for simulation models and can provide ranges of possible values that can help parameter estimation. Based on our results, we discuss future avenues and challenges for modeling AIV epidemics and evaluating control strategies.

## Materials and methods

This systematic review was conducted in compliance with the guidelines of Preferred Reporting Items for Systematic Reviews and Meta-Analysis (PRISMA) [33].

### Search strategy

Three online databases (PubMed, Web of Science, and CAB Abstracts) were searched for relevant literature on mechanistic models used to study the transmission of avian influenza in domestic poultry populations. Three groups of terms were used for each database and linked with the “*AND*” Boolean; within each group, we used an association of keywords linked with the “*OR*” Boolean (Additional file 1). All searches were done in the article title, abstract, and keywords. Articles in languages other than English were excluded. The last search was performed on April 18, 2023.

### Inclusion and exclusion criteria

The final list of articles to include in the review was defined through a two-step screening process (Additional file 2). In the first step, titles and abstracts were independently screened by two reviewers (BB and AM) based on four criteria. Articles were included if they: (1) were primary research articles on avian influenza, (2) focused at least in part on domestic poultry populations, (3) described the propagation of avian influenza epidemics at the population level, and (4) described a mechanistic approach for modeling the spread of avian influenza. Based on these criteria, editorials, commentaries, reviews, and perspective articles were excluded. Articles without any explicit description of transmission processes, or presenting only experimental or molecular data, also were excluded. During this step, a conservative approach was taken, with all articles selected by at least one of the reviewers kept for the next step.

The second screening step was carried out based on the full-text content. Articles were included if they met the inclusion criteria of the first screening, and if the model described was used to achieve at least one of the following two objectives: (1) estimation of model parameters using avian influenza epidemic data, and (2) evaluation of control strategies using a model fitted to field data in the same or in a previous study. Articles that focused solely on simulated epidemics were therefore excluded (i.e., at least one parameter of the model had to be estimated by fitting the model to observed data; models where all parameter values were assumed were excluded). We also looked at the reference lists of the articles included to find further articles that may have been missed in the primary search. Finally, both reviewers discussed their respective final selections until a consensus was reached on each article. In the absence of consensus, the opinion of a third reviewer (SL) was sought.

### Data extraction and analysis

The first three authors worked independently to systematically record the key features of all selected articles. The information extracted included (Additional file 3): contextual information (year of the epidemic(s) studied, poultry population, AIV subtype, virus pathogenicity, geographical location, scale), control strategies (surveillance and control measures evaluated), modeling approach (modeling aim, model paradigm, epidemiological unit, contact structure, transmission routes) and transmission parameters estimated. For within-farm models, when the value of the basic reproduction number *R*_0_ was not reported, it was calculated by multiplying the value of the transmission rate *β* by the value of the average duration of the infectious period, wherever possible. All extracted data were checked for consistency by the first two authors. Descriptive statistics and figures were done using R software [34].

## Results

### Included articles and epidemiological characteristics

**Figure 1:**
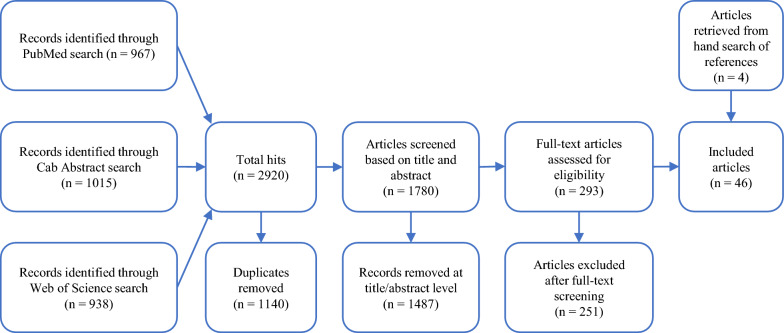
**PRISMA flow diagram of the article selection process**.

Based on an initial search on April 24, 2019 and two search updates on October 21, 2021 and April 18, 2023, the search query yielded 2920 articles, of which 1140 were duplicates (Figure 1). Of the 1780 remaining articles, 1487 were removed after the first screening and 251 after the second. Four additional articles which met the inclusion criteria were identified by checking the references of the articles that passed both screenings. As a result, a total of 46 articles were included in the review [35–80] (Figure 1).

**Figure 2:**
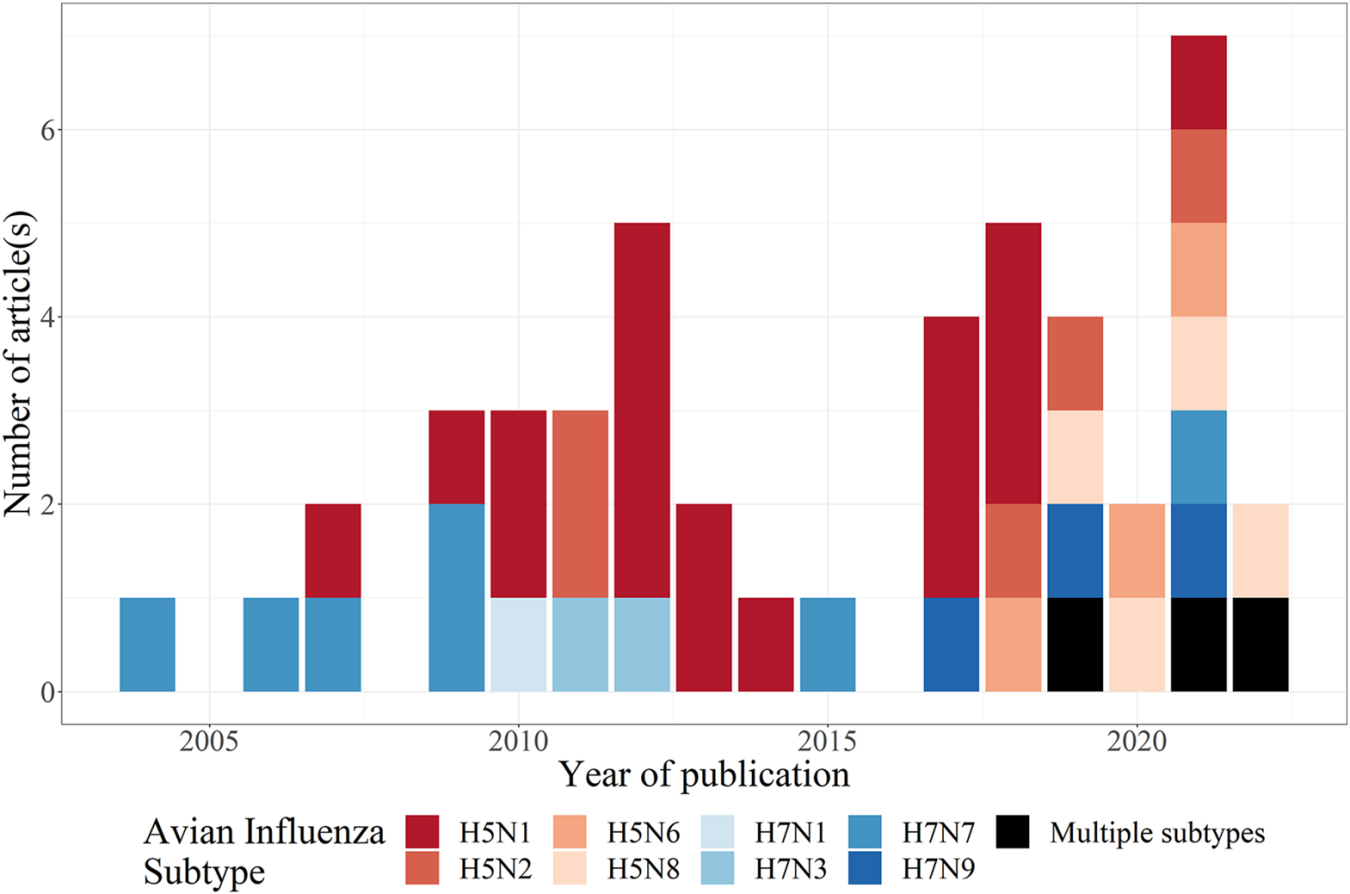
**Temporal description—distribution of publication date for different avian influenza subtypes**. Kim and Cho [43], Lee et al. [60] and Chen et al. [66] studied multiple H5 subtypes.

The epidemiological context of the models presented in these articles varied in terms of pathotype (LPAI or HPAI), subtype, and location (Table 1, Figure 2). The vast majority of articles focused on HPAI (*n* = 41), with three studies on LPAI and two on both HPAI and LPAI simultaneously (Table 1).

**Table 1:** **Overview of the 46 articles included in the review**.

Pathotype	Subtype	Country	Epidemic years	References
HP	H5N1	Thailand	2004–2005	[35-38]
			2004–2007	[39]
		Vietnam	2004–2005, 2007	[40]
			2008–2015	[41]
		South Korea	2008	[42]
			2003–2004, 2008, 2010–2011	[43]
		Indonesia and global	2008–2001	[44]^a^
		Global	2005–2009	[45]^a^
		Bangladesh	2007–2012	[46]^a^; [48]
			2007–2008, 2011	[47]
		India	2008–2010	[49]
		Nigeria	2006–2007	[50]
			2005–2008	[51]
		Egypt	2010	[52]^a^
		Romania	2005–2006	[53]
	H5N2	United States	1983–1984	[54, 55]
			2015	[56]; [57]
	H5N6	Philippines	2017	[58]^a^; [59]
		South Korea	2016	[60]
			2016–2018	[43]
			2016–2017	[61]
	H5N8	South Korea	2016	[60]
			2014–2015, 2016–2018	[43]
		Netherlands	2014, 2016	[62]
		France	2016–2017	[63]
			2020–2021	[64]
		Japan	2020–2021	[65]
	H5Nx	China	2005–2019	[66]
	H7N1	Italy	1999–2000	[67]
	H7N3	Canada	2004	[68]
	H7N7	Netherlands	2003	[69-75﻿]
LP	H5N2	United States	2018	[76]
	H7N3	Netherlands	2003	[77]
	H7N9	China	2013–2015	[78]^a^
LP and HP	H7N9	China	2013–2017	[79]^a^; [80]^a^

^a^Articles in which zoonotic transmission (from birds to humans) was considered.

The majority of articles (*n* = 18; Table 1, Figure 2) focused on the HPAI H5N1 subtype. Although HPAI H5N1 has spread globally since its emergence in 1996, it is noteworthy that models were applied to only a limited number of countries. Thirteen articles analyzed HPAI H5N1 spread in Asia (Thailand, Vietnam, Bangladesh, India, Indonesia and South Korea), three in Africa (Nigeria and Egypt), one in Europe (Romania), and two at the global scale.

The second most studied subtype was HPAI H7N7 (*n* = 7; Table 1, Figure 2). All seven articles, including the earliest modeling article published in 2004 [69], investigated the 2003 HPAI H7N7 epidemic in the Netherlands.

Other past HPAI epidemics analyzed so far included H5N8 in Japan, France and the Netherlands (*n* = 4), H5N2 in the United States (*n* = 4), H5N6 in South Korea and the Philippines (*n* = 3), H7N3 in Canada (*n* = 1), H7N1 in Italy (*n* = 1), and multiple H5 subtypes in South Korea and China (*n* = 3).

Three studies focused on LPAI viruses (Table 1): LPAI H5N2 in the United States (*n* = 1), LPAI H7N3 in the Netherlands (*n* = 1) and LPAI H7N9 in China (*n* = 1). Finally, two studies focused on both LPAI and HPAI H7N9 in China.

### Modeling approaches

In accordance with our inclusion criteria, the most frequent modeling objective was to estimate model parameters (45/46), followed by the evaluation of control strategies (20/46), with 19 articles doing both. Only one article evaluated control strategies without estimating model parameters: Hill et al. [47] re-used the model fitted to epidemic data in Hill et al. [46] to evaluate control strategies in the same epidemiological context.

Most articles included a stochastic component (33/46), while the others were deterministic (13/46). Two different modeling approaches (Box 1) were used in the selected articles: population-based models (PBM, 27/46) and individual-based models (IBM, 19/46; Table 2). All PBMs assumed homogeneous mixing between epidemiological units (i.e., where one epidemiological unit could contact any other unit in a population randomly with equal probability). Most of the IBMs (15/19) assumed that effective contacts depended on the distance between two epidemiological units; either contacts occurred with equal probability but only with units within a given radius, or contacts could occur with any other unit but with a probability that decreased with increasing distance. One IBM used a network to represent contact between farms through recorded vehicle movements [61]. Finally, three IBMs considered transmission at two levels, with homogenous mixing of the epidemiological units (individuals, farms) within larger subpopulations (farms, areas), and distance-dependent contacts between these subpopulations [38, 60, 74]. In these three cases, the “individuals” in the IBMs were the subpopulations and not the epidemiological units.

**Box 1:** Definitions (see [90, 97–99])

**Population-based models**: although the overall population is made up of individual units, population-based models group all individual units of the same state together, without distinction between individual units belonging to the same subgroup. The number (or proportion) of the epidemiological units in each subgroup (e.g., susceptible, infectious, recovered) is tracked, but not the individual state of each unit (for instance, we know how many individual units are infectious, but not which ones). These models are also called compartmental models. **Individual-based models**: contrary to population-based models, individual-based models monitor explicitly the state of each individual unit in the overall population. Therefore, it is possible to track both which individual units are in each state, and also the number of epidemiological units in each state. **Epidemiological unit**: the unit of interest and the smallest entity of the model. It could be an individual animal, a group of animals, herds, or populations in regions or countries. The epidemiological unit can be aggregated and modeled as a number or proportion of the overall population in each state, or modeled as individuals whose status is tracked. **Frequency-dependence** (sometimes referred to as “pseudo-mass action”): with this assumption, the contact rate (the number of contacts made by each epidemiological unit per unit time, where contacts are of an appropriate type for transmission to be possible) is constant irrespective of the population size $N\left(t\right)$. In that case, the force of infection is of the form $\beta \frac{I\left(t\right)}{N\left(t\right)}$, where $\beta$ is the effective contact rate (in time^−1^), i.e., the number of effective contacts made by each epidemiological unit per unit time. An effective contact is a contact that would effectively lead to transmission if the contact is between an infectious and a susceptible unit. **Density-dependence** (sometimes referred to as “mass action”): with this assumption, the contact rate increases with the population size $N\left(t\right)$. In that case, the force of infection is of the form $\beta I\left(t\right)$, where $\beta$ (in unit^−1^.time^−1^) is the *per capita* rate at which two specific epidemiological units come into effective contact per unit time. Note that the density-dependent $\beta$ is not equivalent to the frequency-dependent $\beta$ because of these different assumptions on the contact rate.	

Three types of epidemiological units were considered: individual birds, poultry farms, and geographical areas defined by administrative boundaries (e.g., villages, districts, counties, countries). When considering individuals as epidemiological units (17/46 articles, Table 2), the health statuses of birds were classically defined as susceptible (S), exposed—infected but not yet infectious (E), infectious (I), recovered (R) or dead (D). SID (*n* = 10) and SEIRD (*n* = 4) models were used most frequently. When considering farms as epidemiological units, the whole farm was considered as exposed and then infectious after the onset of infection (i.e., neglecting within-farm dynamics). Similarly, for administrative areas, the whole area was considered as exposed when at least one outbreak (e.g., one infected farm) occurred, and then the whole area became infectious at the end of the latent period. At these levels (farms, areas), the recovered state was not considered; after being infectious, the whole epidemiological unit was either completely depopulated, or completely susceptible again. At the farm and administrative area levels (29/46, Table 2), the models used most frequently were SEID (*n* = 16) and SID (*n* = 9).

**Table 2:** **Model paradigm, epidemiological unit and transmission scale of the 46 articles included in the review**.

Model paradigm	Pathotype	Epidemiological unit	Scale of transmission	References
Population-based model (*n* = 27)	HP	Administrative area (e.g., village, district, region, country…) (*n* = 4)	Between-areas (*n* = 4)	[37, 49, 51, 53]
		Farm (*n* = 7)	Between-farms (*n* = 7)	[39, 41, 43, 48, 68, 69, 72]
		Individual birds (*n* = 11)	Within-area (*n* = 5)	[44, 45, 52, 58, 66]
			Within-farm (*n* = 6)	[35, 57, 62, 64, 65, 73]
	LP	Individual birds (*n* = 3)	Within-area (*n* = 1)	[78]
			Within-farm (*n* = 2)	[76, 77]
	LP and HP	Individual birds (*n* = 2)	Within-area (*n* = 2)	[79, 80]
Individual-based model (*n* = 19)^a^	HP	Administrative area (e.g., village, district, region, country…) (*n* = 4)	Between-areas (*n* = 4)	[36, 40, 50, 55]
		Farm (*n* = 16)	Between-farms (*n* = 14)	[42, 46, 47, 50, 54–56, 59, 61, 63, 67, 70, 71, 75]
			Between-farms and between-areas (*n* = 2)	[38, 60]
		Individual birds (*n* = 1)	Within-farm and between-farms (*n* = 1)	[74]

^a^The total of individual-based models is not the sum of individual-based models with different epidemiological units because Pelletier et al. [50] and Rorres et al. [55] each developed two models, one using administrative area as the epidemiological unit and one using farm as the epidemiological unit.

Most of the PBMs presented in the review (16/27; Table 2) defined individual birds as the epidemiological unit. Seven studied the transmission dynamics between individual birds and humans [44, 45, 52, 58, 78–80], eight between individual birds in a farm [35, 57, 62, 64, 65, 73, 76, 77], and one between domestic birds and wild birds [66]. The other PBMs (11/27; Table 2) studied transmission between poultry farms [39, 41, 43, 48, 68, 69, 72] or transmission between poultry populations pooled at the administrative unit level [37, 49, 51, 53].

Most IBMs (16/19; Table 2) investigated the transmission process between farms [42, 46, 47, 50, 54–56, 59, 61, 63, 67, 70, 71, 75] or between administrative areas [36, 40, 50, 55], with two articles evaluating both in the same study [50, 55]. The remaining three articles considered transmission at two different scales (e.g., within-farm and between-farms) [38, 60, 74].

Data on poultry species explicitly included in the model were specified in only 20 of the 46 articles. The data mainly covered chickens (*n* = 17), ducks (*n* = 9) and turkeys (*n* = 7). Four articles included other species, such as geese, quails, and ostriches [36, 61, 63, 67]. Even when demographic data were available on various poultry species, poultry populations usually were considered to be one homogeneous population. There were a few exceptions that distinguished backyard from commercial farms [36, 68, 72], or that incorporated the heterogeneity in transmission between host species and sometimes estimated their relative contribution to transmission [36, 38, 60, 63, 67].

Similarly, heterogeneity in transmission routes was rarely considered. Most transmission models considered that AIV transmission occurred only via poultry-to-poultry transmission, with only 10 of the 46 considering other transmission routes (e.g., contacts with wild birds or contaminated environment). For example, some authors modeled explicitly the environment [45, 66, 79] or the disease dynamics in wild birds [36, 45, 49, 66], while others considered a constant, distance-independent parameter to implicitly capture other undefined transmission sources (e.g., infectious backyard farms or wild birds, long-distance movements of infected birds or contaminated equipment) [38, 46, 47, 56, 63].

### Parameter estimations

The between-individual transmission rate $\beta$ was systematically estimated in all nine articles considering within-farm transmission (eight PBMs and one IBM; Table 3). Although all nine models assumed homogeneous mixing and frequency-dependent contact rates, the values of the between-individual transmission rate ranged from 0.6 to 34.4 per day for HPAI viruses, and from 0.5 to 3.9 per day for LPAI viruses (Table 3). Accordingly, values for the between-individual basic reproduction number *R*_0_ ranged widely, from 2.18 to 86 for HPAI viruses, and from 4.7 to 45.942 for LPAI viruses (Table 3). The most common parameters other than *β* and *R*_0_, i.e., the average durations of the latent and infectious periods and the case fatality risk, were most often fixed using published values from infection experiments (Additional file 4). The average duration of the latent period was always less than two days, irrespective of subtype and pathogenicity, and the average duration of the infectious period ranged from 1 to 15 days (Additional file 4). The case fatality risk ranged from 20 to 100% for HPAI viruses, while it was very low (0–1%) for LPAI viruses. Five of the nine articles also estimated the time of virus introduction in addition to the above-mentioned parameters [62, 64, 65, 76, 77]. All parameter values in within-farm transmission models of HPAI viruses were estimated using daily recorded mortality data, while egg production data [77] or diagnostic testing data [76] were used for LPAI viruses (Table 3).

**Table 3:** **Estimated parameter values in within- and between-farm transmission models**.

Reference	Subtype	Model	Transmission rate ${\varvec{\beta}}$	Reproduction number
Within-farm transmission				
[35]	HP/H5N1	PBM/FD	Min: 0.60 (0.43–0.84) Max: 2.30 (1.92–2.76)	Min: 2.18 (1.94–2.46) Max: 3.49 (2.70–4.50)
[57]	HP/H5N2	PBM/FD	3.2 (2.3–4.3)	12.8 (9.2–17.2)
[62]	HP/H5N8	PBM/FD	Min: 0.95 (0.3–2.3) Max: 34.4 (27.3–44.1)	Min: 5.225 (1.65–12.65) Max: 86 (68.25–110.25)
[64]	HP/H5N8	PBM/FD	4.1 (2.8–5.8)	17.5 (9.4–29.3)
[65]	HP/H5N8	PBM/FD	Min: 0.661 (0.627–0.696) Max: 3.387 (1.774–2.071)	Min: 2.642 (2.507–2.785) Max: 13.548 (12.832–14.304)
[73]	HP/H7N7	PBM/FD	4.50 (2.68–7.57)	–
[74]	HP/H7N7	IBM/FD	1.9 (0.61–8.1)	7.6 (2.44–32.4)
[76]	LP/H5N2	PBM/FD	Min: 0.6 (0.4–1.0) Max: 3.9 (1.2–5.8)	Min: 7.068 (4.712–11.78) Max: 45.942 (14.136–68.324)
[77]	LP/H7N3	PBM/FD	Min: 0.50 (0.45–0.55) Max: 0.72 (0.68–0.77)	Min: 4.7 (3.0–8.6) Max: 5.6 (4.3–7.7)
Between-farm transmission				
[38]	HP/H5N1	IBM/DD	0.99 (0.76–1.12) × 10^–6^	–
[41]	HP/H5N1	PBM/DD	Min: 1.4 × 10^–8^ Max: 40 × 10^–8^	Min: 0.55 Max: 15.7
[42]	HP/H5N1	IBM/DD	0.429 (probability)	–
[43]	HP/H5N1 HP/H5N8 HP/H5N6	PBM/DD	–	Min: 0.03 (0–0.98) Max: 2.20 (1.51–3.16)
[46, 47]	HP/H5N1	IBM/DD	Min: 1.71 (0.586–3.63) × 10^–10^ Max: 1.06 (0.0729–3.78) × 10^–7^	–
[48]	HP/H5N1	PBM/FD	Min: 0.08 (0.06–0.10) Max: 0.11 (0.08–0.20)	Min: 0.85 (0.77–1.02) Max: 0.96 (0.72–1.20)
[56]	HP/H5N2	IBM/DD	0.0061 (0.0025–0.0137)	–
[59]	HP/H5N6	IBM/DD	0.0012 (0.0001–0.1)	–
[60]	HP/H5N6 HP/H5N8	IBM/DD	Min: 0.00007–Max: 0.00707	–
[63]	HP/H5N8	IBM/FD	Min: 0.23 (0.16–0.31) Max: 0.53 (0.37–0.72)	–
[67]	HP/H7N1	IBM/DD	Min: 0.0009 (0.0005–0.0013) Max: 0.0155 (0.0078–0.0232)	–
[68]	HP/H7N3	PBM/DD	Min: 0 – Max: 0.00238	4.8
[69]	HP/H7N7	PBM/DD*	Min: 0.17 (0.1–0.2) Max: 0.47 (0.3–0.7)	Min: 1.2 (0.6–1.9) Max: 6.5 (3.1–9.9)
[70]	HP/H7N7	IBM/DD	Min: 0.076 Max: 0.336	–
[71]	HP/H7N7	IBM/DD	0.002 (0.0012–0.0039)	–
[72]	HP/H7N7	PBM/DD	1.7 (1.5–2.0) × 10^–4^	1.33
[74]	HP/H7N7	IBM/DD	0.0039 (0.0023–0.0076)	–

FD: frequency-dependence ($\beta$ is in day^−1^); DD: density-dependence ($\beta$ is in farm^−1^.day^−1^).

This model assumed a force of infection of the form $\beta \frac{I\left(t\right)}{N}$, where $N$ was a constant (the initial population size). Although assuming a density-dependent contact rate [69], the unit of $\beta$ was the same as for frequency-dependent models, here in day^−1^.

Values of the reproduction number were not indicated when the average duration of the infectious period was not provided, or when the transmission between farms was distance dependent (because the reproduction number is farm specific).

Similarly, the between-farm transmission rate *β* and the between-farm reproduction number *R*_h_ were the most commonly estimated parameters in the 24 articles studying between-farm transmission of HPAI (Table 3). The average duration of the infectious period at the farm level was the third most frequently estimated parameter, and ranged quite widely depending on epidemiological settings and virus subtypes. However, the smallest estimated value was 6.4 days, indicating rather long infectious periods at the farm level (Additional file 5).

**Figure 3:**
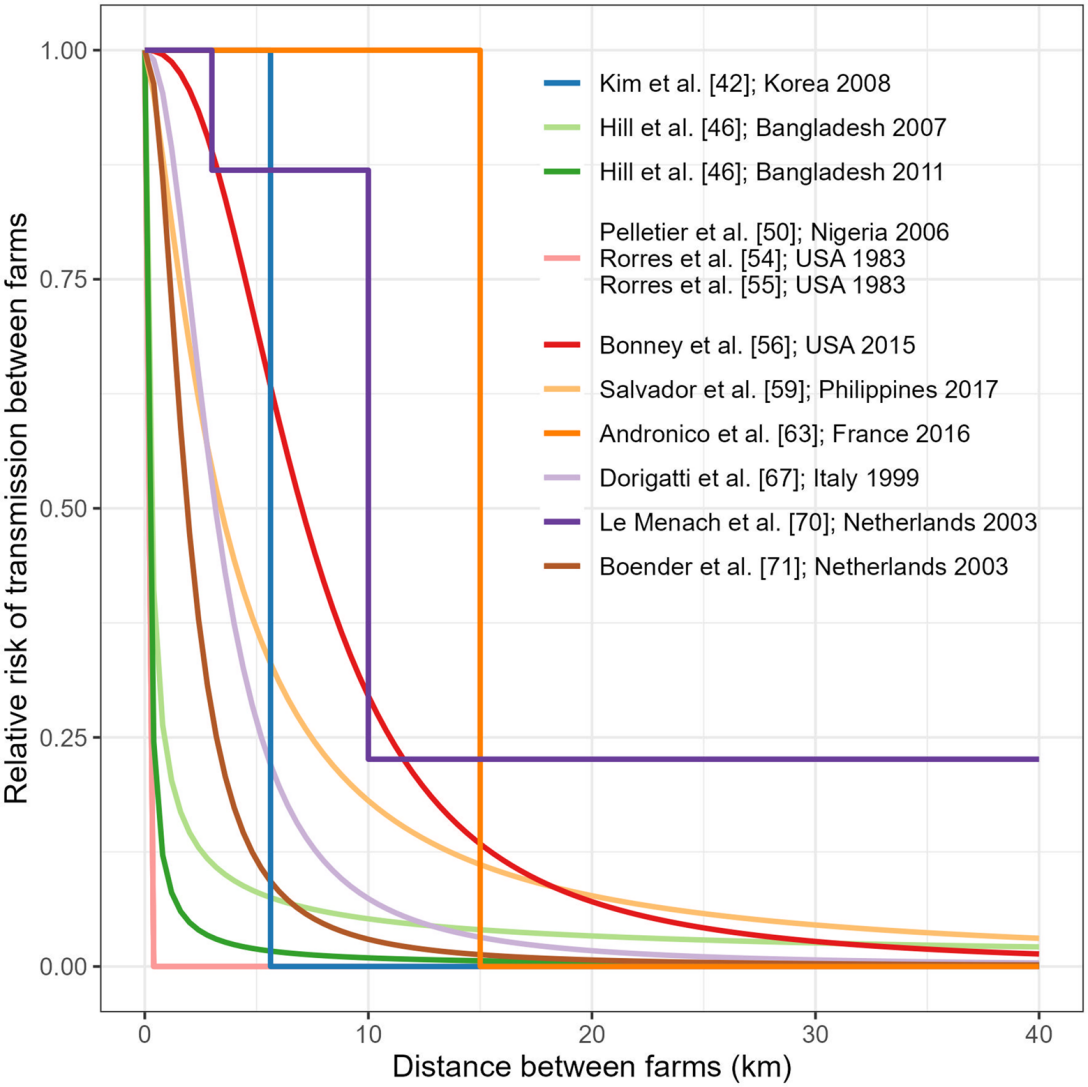
**Comparison of spatial transmission kernels used in between-farm transmission model**. Hill et al. [47] and Backer et al. [74] used the same kernel and same parameter values as Hill et al. [46] and Boender et al. [71], respectively. Note that for Rorres et al. [54, 55] and Pelletier et al. [50], estimated parameter values were close and therefore the three curves are indistinguishable. For these three models, although the relative risk of transmission between farms decreased to very low values at small distances, the absolute probability of transmission remained substantial even at long distances because the sizes of both susceptible and infectious farms were included in the force of infection.

In addition, 14 of the 24 articles modeling between-farm transmission used spatial transmission kernels to describe how the relative risk of transmission between farms changed with the distance between a susceptible and an infectious farm. The resulting spatial transmission kernels are illustrated in Figure 3, except for that of Seymour et al. [75] who used a nonparametric transmission kernel that could not be reproduced. Two studies used a step function where the relative risk was one below a certain distance threshold, and zero otherwise [42, 63]. Le Menach et al. [70] estimated three transmission rates for pre-determined ranges of between-farms distances (less than 3 km, between 3 and 10 km, and more than 10 km), which we converted into a step-function kernel (Figure 3). The other studies used parametric kernels, with three different functions (Table 4):

- a Pareto distribution [46, 47]: $$\left\{\begin{array}{c}1, 0\le {d}_{ij}<{x}_{min}\\ {\left(\frac{{x}_{min}}{{d}_{ij}}\right)}^{\alpha +1}, {d}_{ij}\ge {x}_{min}\end{array}\right.$$
- a power-law function [50, 54, 55]: $1-{e}^{-{\left(\frac{\delta }{{d}_{ij}}\right)}^{\rho }}$
- or a logistic expression [56, 59, 67, 71, 74]: $\frac{1}{1+{\left(\frac{{d}_{ij}}{{r}_{0}}\right)}^{\alpha }}$

**Table 4:** **Estimates of spatial kernel parameters used in between-farm transmission models of highly pathogenic avian influenza**.

Spatial kernel $K\left({d}_{ij}\right)$	Reference	Parameters	
Pareto distribution $$\left\{\begin{array}{c}1, 0\le {d}_{ij}<{x}_{min}\\ {\left(\frac{{x}_{min}}{{d}_{ij}}\right)}^{\alpha +1}, {d}_{ij}\ge {x}_{min}\end{array}\right.$$	[46, 47]	${x}_{min}=0.1 \mathrm{km}$	$\alpha =-0.358 \left(-0.666,-0.159\right)$
			$\alpha =0.0136$ $\left(-0.122, 0.143\right)$
Power-law $1-{e}^{-{\left(\frac{\delta }{{d}_{ij}}\right)}^{\rho }}$	[54]	$\delta =1.529\times {10}^{-5} \mathrm{km}$	$\rho =2.2$
	[55]	$\delta =9.334\times {10}^{-5} \mathrm{km}$	$\rho =2.5$
	[50]	$\delta =5.899\times {10}^{-5} \mathrm{km}$	$\rho =2.009$
Logistic expression $\frac{1}{1+{\left(\frac{{d}_{ij}}{{r}_{0}}\right)}^{\alpha }}$	[71]; [74]	${r}_{0}=1.9 \mathrm{km}$ $\left(1.1, 2.9\right)$	$\alpha =2.1 \left(1.8, 2.4\right)$
	[67]	${r}_{0}=3.160 \mathrm{km}$ $\left(1.770, 4.549\right)$	$\alpha =2.192 \left(1.894, 2.490\right)$
	[56]	${r}_{0}=7.02 \mathrm{km}$ $\left(3.07, 16.16\right)$	$\alpha =2.46 \left(1.80, 4.38\right)$
	[59]	${r}_{0}=3.4 \mathrm{km}$ $\left(1.001, 10.0\right)$	$\alpha =1.4$ $\left(1.001, 5.0\right)$

For estimated parameter values, the mean/median and 95% confidence/credible interval (when reported) are indicated. ${d}_{ij}$ is the distance between an infectious farm $i$ and a susceptible farm $j$. In the Pareto distribution, ${x}_{min}$ is the minimum possible value of the function and $\alpha \ge -1$ determines the shape of the kernel: $\alpha =-1$ corresponds to distance-independent transmission, with increasing values of $\alpha$ increasing local transmission and diminishing long-range transmission. In the Power-law function, the distance-scaling parameter $\delta$ is the distance at which the relative risk of transmission is $1-\frac{1}{e}$ (or about 0.63), and $\rho$ determines whether the decrease from probability 1 to 0 is gradual (small $\rho$) or step like (large $\rho$). In the logistic expression, the half-kernel distance ${r}_{0}$ corresponds to the distance at which the relative risk of transmission is 0.5, and the shape parameter $\alpha$ determines whether the decrease from probability 1 to 0 is gradual (small $\alpha$) or step like (large $\alpha$).

The parameters of these kernels were all estimated (Table 4), except for Backer et al. [74] and Hill et al. [47], who used the same parameter values as Boender et al. [71] and Hill et al. [46], respectively. Rorres et al. [54, 55] and Pelletier et al. [50] estimated large values of the shape parameter *ρ* and very small values of the distance-scaling parameter *δ* (Table 4), thus producing a step-like function where the relative risk of transmission between farms decreased sharply to very small values (Figure 3). However, in these articles, the force of infection accounted for the number of birds in the susceptible and infectious farms, meaning that the probability of transmission between farms remained substantial even at high distances. Hill et al. [46] also found a rapidly decreasing probability, but with long right-tails depending on the epidemic used to parameterize the kernel (Figure 3; Table 4). Similar values of the shape parameter *α* were found in Boender et al. [71], Dorigatti et al. [67] and Bonney et al. [56], thus producing similar shapes. However, they estimated different values of the half-kernel distance *r*_0_, with local transmission being more important in Boender et al. [71] and Dorigatti et al. [67], while transmission at moderate distances remaining substantial in Bonney et al. [56]. Conversely, Salvador et al. [59] found a similar half-kernel distance *r*_0_ to Dorigatti et al. [67], but a smaller shape parameter *α*, indicating a larger role of long-distance transmission (Figure 3, Table 4).

Finally, the remaining 14 articles considered transmission within and/or between areas, with individuals or administrative areas as epidemiological units (i.e., not modeling farms). Eleven estimated both the transmission rate and the* R*_h_, three estimated the average duration of the infectious period, and two estimated the average duration of the incubation period and spatial kernel parameters. However, variations between the epidemiological units and spatial scales considered limit comparisons and the possibility to use values for different settings. Therefore, we did not report the estimated values here (but they are presented in Additional file 3).

### Mitigation strategies

Out of the 46 articles selected, 20 evaluated the effectiveness of control measures in containing AIV outbreaks through numerical simulations (Table 5). Most focused only on HPAI outbreaks, with one exception that focused on both LPAI and HPAI H7N9 in China (Table 5). Five studies used PBMs and 15 used IBMs. Most articles (15/20) studied the effect of control measures solely on epidemiological parameters in the poultry population (epidemic size and duration, reproduction number, number of culled flocks). The economic impact of the measures implemented was considered in only three articles [38, 74, 75]. Finally, in two studies [52, 80], the authors estimated the effectiveness of mitigation strategies in poultry for controlling the disease in humans (e.g., reduction of the number of infected humans).

**Table 5:** **Mitigation strategies evaluated in articles included in the review**.

Reference	Pathotype	Outcome for comparison^a^	Mitigation strategies evaluated^b^				
			RC	PEC	Vacc	Surv	Others
[36]	HP	Epi				x	
[38]	HP	Epi + Eco		x	x		
[40]	HP	Epi			x	x	
[42]	HP	Epi		x		x	
[47]	HP	Epi		x	x	x	
[49]	HP	Epi	x				
[50]	HP	Epi		x	x		
[52]	HP	Epi H	x				
[56]	HP	Epi					Early marketing
[58]	HP	Epi			x		Quarantine
[59]	HP	Epi		x		x	
[60]	HP	Epi		x			
[63]	HP	Epi	x	x		x	
[66]	HP	Epi	x				Biosecurity measures and closure of live poultry markets
[67]	HP	Epi		x			Ban on restocking
[70]	HP	Epi	x	x		x	National movement ban
[71]	HP	Epi		x			
[74]	HP	Epi + Eco		x	x		
[75]	HP	Epi + Eco		x			
[80]	LP and HP	Epi H					Biosecurity measures and closure of live poultry markets

^a^Outcome considered by the authors to compare mitigation strategies: Epi, epidemiological parameters in poultry; Eco, economic impact of measures evaluated; Epi H, epidemiological parameters in humans.

^b^Mitigation strategies evaluated: RC, reactive culling; PEC, pre-emptive culling; Vacc, vaccination; Surv, surveillance.

The most commonly evaluated strategy was poultry culling (15 articles, Table 5), either in the form of reactive culling (culling of infected flocks, five articles) or pre-emptive culling (culling of at-risk flocks not necessarily infected, 12 articles), with two articles evaluating both.

Reactive culling was most of the time included in the model baseline mitigation strategy without evaluating its effectiveness on its own [36, 38, 40, 47, 49, 50, 56, 59, 60, 63, 67, 70, 71, 74, 75, 80]. There were two exceptions: (1) Lee et al. [52] showed that reducing the number of infectious poultry led to a substantial reduction in the number of H5N1 infections in humans compared to a scenario without reactive culling; and (2) Chen et al. [66] showed that reactive culling could substantially decrease the number of poultry outbreaks, but could not control the epidemic on its own. In addition, the impact of the timeliness of reactive culling was evaluated in three articles, which all consistently showed a strong positive impact of this parameter on the effectiveness of reactive culling [49, 63, 70]. For example, Andronico et al. [63] showed that reducing the time between detection and culling (from 5 to 2 days) cut in half the expected size of the outbreak in poultry.

Pre-emptive culling consisted of culling flocks within a given radius around detected infected flocks [38, 47, 50, 59, 60, 63, 67, 70, 71, 74, 75]. The radius was under 10 km in most articles, except for Kim et al. [42], who evaluated radii up to 56.25 km. All articles reported the effectiveness of pre-emptive culling on reducing the number of infected flocks and the duration of the epidemic. The effectiveness increased when the culling radii increased. However, the economic costs of the epidemic were also the highest for the highest culling radii [74, 75].

Moreover, the improvement brought by increasing the pre-emptive culling radius saturated rapidly when limited culling capacity was considered [47, 50, 60, 63, 70, 74, 75]. This is because further increasing the culling radius did not change the number of culled flocks once the maximum culling capacity was reached. Limited culling capacity was introduced to represent limited resources and logistical difficulties presented by mass culling over a short period of time. It was modeled either as a number or proportion of preventively culled farms around each infected farm [63, 70], as a maximum number of farms culled at each time step [47, 50, 74, 75], or as a nonlinear culling rate depending on the number of reported farms [60]. Increasing the culling capacity substantially increased the effectiveness of pre-emptive culling on the size and duration of an epidemic [47, 74].

The effectiveness of pre-emptive culling came at the cost of culling more flocks. In most cases, the total number of culled flocks (by reactive and pre-emptive culling) increased as the pre-emptive culling radius increased. This means that reactive culling without pre-emptive culling would be preferred if the objective was to reduce the number of culled flocks instead of reducing the epidemic size [38, 47, 63, 67, 74, 75]. However, in some cases the total number of culled flocks was non-monotonic as the pre-emptive culling radius increased [42, 59, 60]. The total number of culled flocks first decreased, as the number of infected flocks decreased faster than the number of pre-emptively culled flocks increased. The total number of culled flocks then increased again as there were more and more pre-emptively culled flocks. Therefore, there was a minimum number of culled flocks at an optimal radius, which ranged from 1 to 18.75 km depending on the epidemiological setting [42, 59, 60]. In Lee et al. [60], this non-monotonic behavior was only observed in areas with *R*_0_ above one, but not in areas with *R*_0_ below one.

Vaccination strategies were evaluated less frequently than culling strategies (six articles; Table 5). Vaccination was applied either within a radius of 1 to 10 km around infected flocks [47, 50, 74] or to a proportion of the total poultry population [38, 40, 50, 58]. One article evaluated the impact of a vaccination strategy that was applied in real life and not as a theoretical scenario [40]. The results showed that the countrywide vaccination strategy implemented in Vietnam starting in 2005 with ~60% coverage allowed the transmissibility of HPAI infection to be substantially reduced. However, this was coupled with a substantial increase of the time from infection to detection, possibly because lower levels of mortality and symptomatic infections made outbreaks harder to detect [40]. When considering hypothetical vaccination strategies, the results showed the effectiveness of vaccination from an epidemiological point of view in most articles, although ring vaccination was found ineffective in some cases [47, 50]. Pelletier et al. [50] showed that ring vaccination was less effective than countrywide vaccination with 92–97% coverage. The effectiveness of countrywide vaccination was further improved when the order in which premises were vaccinated was determined by known risk factors, such as flock size or proximity to an infected flock [50]. Increasing the vaccination radius brought limited improvements when considering limited capacity [47].

Vaccination was compared to pre-emptive culling in four articles [38, 47, 50, 74]. Their respective effectiveness depended on the objective behind their implementation: vaccination was more effective in reducing the number of culled flocks, while pre-emptive culling was more effective in reducing epidemic size and duration [38, 47, 50, 74]. In Pelletier et al. [50], countrywide vaccination was the most effective in reducing the number of culled flocks, while at the same time having similar effectiveness as pre-emptive culling in reducing the epidemic size. However, the effort required by these two control strategies was substantially different: 92 to 97% of flocks were vaccinated while only 17 to 27% were culled [50]. Increasing the management capacity was less effective for ring vaccination than for ring culling [47, 74].

Only two studies compared the relative costs of pre-emptive culling and vaccination strategies [38, 74]. Backer et al. [74] found that the duration and size of the epidemics were lower for pre-emptive culling than for ring vaccination for a same radius (3 km). However, the total costs (direct costs—such as compensation of culled poultry, costs of culling, cleaning and disinfecting, costs of vaccine doses and vaccination, and surveillance costs—as well as indirect costs—such as lower prices for eggs and slaughtered poultry, and loss of revenue) of the two strategies were only marginally different. Retkute et al. [38] showed that the choice of a strategy depended on the relative costs of pre-emptive culling and vaccinating compared to the economic impact of an infected flock (e.g., costs of reactive culling, compensation paid for destroyed animals, or costs associated with potential zoonotic transmission to poultry professionals). If the cost of culling was low compared to the cost of an infected flock, pre-emptive culling was preferred regardless of the cost of vaccination. If the costs of culling and vaccinating were both high compared to the cost of an infected flock, then neither strategy were preferred and reactive culling alone minimized the total costs of the epidemic. However, if the cost of culling was high compared to the cost of an infected flock but the cost of vaccination was low, then vaccination was preferred [38].

Seven articles evaluated the effectiveness of surveillance strategies (Table 5). Andronico et al. [63] demonstrated that the surveillance zone implemented as part of the mandatory EU strategy during the 2016–2017 H5N8 epidemic in France was effective in reducing transmission. Walker et al. [36] found that intensive surveillance campaigns reduced the period between infection and reporting during the 2004–2005 H5N1 epidemic in Thailand. The other five articles all showed that improving surveillance to reduce the time between infection and detection had a large effect on the epidemic [40, 42, 47, 59, 70]. For instance, reducing the average duration between infection and detection by 35% reduced the epidemic size by 66.8% in Walker et al. [40]. Hill et al. [47] even concluded that improving surveillance was in most cases a more effective strategy than pre-emptive culling or ring vaccination.

Various other strategies (e.g., movement bans, biosecurity measures, closure of live poultry markets) were evaluated in eight articles (Table 5). Most articles assumed that farms emptied of poultry during an epidemic, either because they had completed a production cycle or because their flocks had been culled, were banned from restocking. However, only Dorigatti et al. [67] demonstrated the effectiveness of this mitigation measure. They showed that if the ban on restocking had not been implemented, the epidemic size would have been 155% times higher on average [67]. During the HPAI H5N2 epidemic in Minnesota (US) in 2015, a strategy called “early marketing” was used [56]. This strategy consisted in sending flocks to slaughter earlier than the normally scheduled date to reduce the density of poultry farms. By sufficiently decreasing densities, it was possible to decrease the reproduction number to below one in a high-density area, thereby reducing virus spread [56]. The authors therefore considered this strategy to be an interesting alternative or complement to conventional pre-emptive culling [56].

Finally, the early implementation of mitigation strategies was always more effective than implementing them later in an epidemic [67, 70, 80]. When considering the impact of mitigation strategies on zoonotic transmission to humans, it was demonstrated that combining measures in poultry with measures in humans was better than implementing measures in poultry alone [52]. Given the potential difficulties of implementing a single strategy at highly effective levels, the joint implementation of two strategies at less effective levels may be both easier and as (or even more) effective, thus representing an interesting alternative [52].

## Discussion

The results presented in this study summarize the state of the art in mechanistic modeling applied to field outbreaks of low pathogenicity and highly pathogenic avian influenza in poultry. Our objectives were to describe the models and their epidemiological context, to list current estimates of AIV transmission parameters, to provide insights on avian influenza transmission and the impact of control strategies, and finally to discuss future avenues for modeling AIV transmission. We limited our analysis to articles describing a model fitted to epidemic data to ascertain the articles’ relevance for this review. The search was restricted to three online databases for articles in English based on a selected set of keywords. Therefore, it is possible that we missed a few relevant publications in the gray literature which did not have the selected keywords or were in a language other than English. However, we are confident that this analysis provides an accurate literature synthesis.

### Epidemiological insights

#### Parameter estimates

Two recent studies reviewed values of the transmission rate, the basic reproduction number, and the average durations of the latent and infectious periods estimated from experimental studies [31, 32]. In addition, Kirkeby and Ward [32] included estimates from field data, but only if no disease control strategies had been implemented when the data used to perform the estimation was collected. Our review of parameter values therefore differed from these previous studies, as we excluded studies based on experimental infections and included all estimates from field data, including those from periods with active disease control strategies. This was in line with our objectives, which were to review both parameter values and effectiveness of disease control strategies estimated using mechanistic models fitted to field outbreaks.

In our study, only two articles estimated the average durations of the latent and infectious periods in individual birds (Additional file 4) [64, 77], preventing comparisons with the previous reviews. In Kirkeby and Ward [32], the average latent period ranged from 0.2 to 2 days for HPAI, and from 0.02 to 8.6 days for LPAI. The average infectious period for HPAI ranged from 1 to 6.8 days in [32], and from 1.47 days to 13.4 days in [31]. For LPAI, the average infectious period ranged from 2.3 to 13.32 days in [32], and from 4.25 to 10.03 days in [31].

As in our study, both of these reviews found a large variation in the estimated between-individual transmission rates and basic reproduction number *R*_0_ [31, 32]. The values of the between-individual reproduction number ranged from 2.18 to 86 for HPAI viruses in our study (Table 3), from 0.17 to 208 in [32] and from 1.6 to 34 in [31]. For LPAI, *R*_0_ ranged from 4.7 to 45.9 in our study (Table 3), from 0.3 to 15.6 in [32] and from 0.22 to 15.3 in [31].

In our review, estimates of the average duration of the infectious period at the farm level ranged between 6.4 and 17.22 days for HPAI (Additional file 5). In comparison, estimated values of this parameter ranged between 7.9 and 13.8 days in Kirkeby and Ward [32]. The between-farm reproduction number *R*_h_ for HPAI ranged from 0.03 to 15.7 in our review (Additional file 5), and from 0.85 to 22.4 in [32].

The estimates presented in the studies selected for the current review were from a wide range of geographical regions and virus subtypes. This will undoubtedly improve risk analysis and the creation of future models by constructing more realistic simulation studies. While some insights may be gained into the virus spread dynamics in these areas, caution should be taken to extrapolate parameters from one region to the next as disease dynamics depend on numerous factors, including local conditions, farm density, and production systems [81].

#### Transmission mechanisms

As illustrated in Figure 3, different transmission models used distance-based kernels to study HPAI spread between poultry farms. The estimated kernel parameters (Table 5) suggest that most HPAI transmissions happened at a short to moderate distance range, irrespective of the subtype or geographical location. This is in line with results from observational studies [27, 82]. However, it does not mean that long-distance transmission is impossible. For example, the spatial kernels used in Bangladesh [46] and the Philippines [59] were long-tailed, indicating that some transmission events were often sourced over long distances. Differences in spatial kernels were observed between epidemics in different countries. Differences also could appear between different epidemics of the same subtype in a given country, as highlighted in Hill et al. [46], or even between different waves in a given epidemic, as suggested in Bonney et al. [56]. Such differences could arise from differences in poultry production, distinct characteristics of different HPAI subtypes, or specific control strategies.

Poultry farming systems vary widely regarding the species raised, farm sizes, and management systems [83]. This heterogeneity likely plays a role in epidemic dynamics, but it was rarely accounted for explicitly. However, when species heterogeneity was explicitly modeled, it was possible to determine the respective contribution of each species to transmission, which appeared to depend on the virus subtype. For example, Andronico et al. [63] explicitly modeled galliformes (e.g., chickens) and palmipeds (e.g., ducks and geese) to account for their contribution to the 2016–2017 HPAI H5N8 propagation dynamics in France. They were able to show that palmiped farms were 2.6 (95% CI: 1.2–10) more susceptible and 5.0 (95% CI: 3.7–6.7) times more infectious than galliform farms. Similar results were found in the Republic of Korea for the 2016–2017 HPAI H5N8 spread, with transmissibility between duck farms being as much as 1.6 times higher than either between chicken farms or between different farm types [60]. In contrast, chicken farms were up to 10.7 times more infectious than palmiped farms during the 2004 HPAI H5N1 epidemic in Thailand [36].

Similarly, heterogeneity between management systems was explicitly accounted for in three articles by considering separate compartments for commercial and backyard poultry farms [36, 68, 72]. Both Bavinck et al. [72] and Smith and Dunipace [68] found that backyard farms played a limited role in HPAI transmission, with reproduction numbers below one for transmission between backyard farms and between commercial and backyard farms (note that Walker et al. [36] used a common compartment for backyard poultry and wild birds, preventing comparison).

Wild birds were sometimes included to account for sources of infection other than domestic poultry farms. Different approaches were used, either by implementing a constant parameter fitted to the epidemic data and implicitly considering various sources of transmission (including wild birds) [38, 46, 56, 63], by including seasonal introductions into poultry farms via migratory birds [45], or by considering a specific compartment for the number of wild birds in the vicinity of domestic farms [36, 49]. When the relative role of wild birds and domestic poultry was assessed, commercial poultry farms were found to be the main sources of infection [45, 49, 63]. For instance, Andronico et al. [63] quantified that wild birds and backyard poultry only accounted for 11% of transmission events. However, wild birds may be playing an important role in introducing and re-introducing the virus to the local environment [45, 49].

#### Control strategies

About half of the included articles evaluated control strategies (20/46). Overall, both reactive and pre-emptive culling remained the most studied control measures, reflecting the widespread use of these measures in real life. In contrast, vaccination was evaluated more rarely. The use of vaccination as a routine component of the control strategy was until 2023 prohibited in the EU [84], and currently remains prohibited in the US [16] because of the trade implications. However, it was authorized in the EU in 2023 [17] due to the recent devastating epidemics.

The effectiveness of control measures likely depends on the virus subtype and on local conditions, such as farm density and the distribution of farm types (backyard/commercial) and poultry species. For instance, the optimal radius minimizing the number of preventively culled flocks varied between locations and epidemic years [42, 59, 60]. Similarly, the optimal strategies minimizing the costs of HPAI epidemics depend on the relative costs of various control measures [38], which may vary over time and between countries. Therefore, whenever possible, modeling approaches should consider the local context and time period when assessing control strategies.

Nonetheless, we identified two general findings regarding the evaluation of control strategies. First, the optimal control measure depended on the objective. The best strategy often differed depending on whether the objective was to reduce the epidemic duration and size or whether it was to reduce the number of culled flocks [38, 47, 50, 74]. In particular, vaccination was often the strategy associated with the lowest number of culled flocks, thus possibly reducing the costs of an epidemic (e.g., median of 278 culled farms for the baseline scenario *vs* only 163 culled farms in the same scenario but with ring vaccination [74]). However, vaccination strategies may incur other costs by increasing epidemic durations (e.g., median of 67 days for ring vaccination compared to a minimum of 26 days for ring culling [74]) or by disrupting international trade. It should be noted that losses due to restrictions on exports were not included in the three models that compared control strategies based on economic costs [38, 74, 75]. Therefore, a careful definition of the objective and costs associated with each measure is necessary to have a more comprehensive evaluation of the cost-effectiveness of each strategy.

Second, the early implementation of control measures supported by effective surveillance was always beneficial. For instance, reactive culling of infected flocks soon after their detection had a substantial impact on the course of HPAI epidemics [49, 63, 70]. Similarly, improving surveillance to reduce the time between infection and detection/reporting, thus allowing earlier implementation of various strategies (e.g., reactive culling of the infected flock, pre-emptive culling around it), substantially reduced the impact of HPAI epidemics [36, 40, 42, 47, 59, 70]. Moreover, the effectiveness of control strategies increased as the period between the first detected outbreak and the first implementation of control measures decreased [67, 70, 80]. For the 1999–2000 HPAI H7N1 epidemic in Italy, the number of outbreaks would have been reduced from 385 to 222 on average if preventive culling had been implemented 20 rather than 54 days after the epidemic started [67]. All of these results highlight the importance of epidemic preparedness, with effective surveillance systems and quick responses of policy makers and veterinary services.

#### Different insights from different modeling approaches?

Different modeling approaches have been used to evaluate AIV transmission during the same epidemics in the same countries (Table 1). This was the case for the 2003 HPAI H7N7 epidemic in the Netherlands [69–72, 74, 75] and for the 2004 HPAI H5N1 epidemic in Thailand [36–38]. In these epidemics, different modeling approaches were applied to the same datasets with varying levels of details, providing varying parameter estimates. The 2003 HPAI H7N7 epidemic in the Netherlands was by far the most studied (Table 1), and provided a good case study of how various modeling approaches influence parameter estimations and recommendations to policy makers. Two population-based models neglected the spatial aspect of between-farm transmission [69, 72]. Contrary to Stegeman et al. [69], Bavinck et al. [72] accounted for backyard flocks and neglected temporal changes in transmission rates and infectious periods during the study period (22 February-3 April 2003). Nonetheless, they estimated a between-farm reproduction number (*R*_h_) of 1.33 (Table 3), quite similar to the 1.2 (95% CI: 0.6–1.9) estimates for the same area between 1 March 2003 and 3 April 2003 in Stegeman et al. [69]. Bavinck et al. [72] had the additional advantage of estimating the contribution of backyard and commercial flocks to transmission. They showed that culling backyard flocks may not be necessary as these flocks only played a minor role [72].

Compared to PBMs, spatial IBMs had the additional ability to produce at-risk maps [71] and to evaluate spatially-explicit control strategies such as preventive culling around infected flocks [70, 74, 75]. Various ways of accounting for spatial heterogeneity in transmission during the 2003 HPAI H7N7 epidemic have been used. Le Menach et al. [70] estimated three transmission rates depending on the distance between farms, and found substantially lower transmission rates for long-distance ranges (above 10 km) than for short and medium-ranges (under 3 km and between 3 and 10 km, respectively – Figure 3). Boender et al. [71] used a parametric kernel with a logistic functional form to represent how the relative risk of transmission decreased with increasing between-farm distance (Figure 3). The same kernel was re-used by Backer et al. [74]. The estimates of the spatial kernel parameters indicated a rapid decrease of the relative risk of transmission with increasing distance, where the risk was already halved at 1.9 km and reached very low values beyond 10 km (Figure 3; [71]). Finally, Seymour et al. [75] developed a nonparametric kernel to represent the same phenomenon but in a more flexible way. The estimated nonparametric kernels were of similar shape and scale as the parametric kernel used in Boender et al. [71], but with a higher degree of uncertainty, especially at lower distances [75]. The authors therefore argued that the assumptions imposed by parametric kernels may underestimate the uncertainty, and that nonparametric kernels may better reflect the actual uncertainty in the data [75].

The last point of comparison is the evaluation of control strategies, and more specifically of pre-emptive culling. Boender et al. [71] concluded that culling farms within a 1 km radius was not very effective in reducing the *R*_h_, but that culling farms within a 5 km radius reduced the *R*_h_ of all farms below one, and thus was fully effective. In contrast, Backer et al. [74] and Seymour et al. [75] both found a strong effect of pre-emptive culling within a 1 km radius on the number of infected farms compared to no pre-emptive culling. This difference may be explained by the way the control strategies were evaluated. In Boender et al. [71], the effectiveness of pre-emptive culling was evaluated on the *R*_h_ of all farms. The other two studies simulated the epidemics under different scenarios and compared the model outputs, such as the number of infected and culled farms. Therefore, the criteria used to evaluate the effectiveness of control strategies need to be chosen carefully. Interestingly, Seymour et al. [75] found that increasing the culling radius beyond 1 km did not substantially reduce the number of infected farms despite a larger number of culled farms. In contrast, Backer et al. [74] found that the effectiveness increased when expanding the radius from 1 to 3 km, but not from 3 to 10 km. This difference in the optimal pre-emptive culling radius (1 vs 3 km) could be explained by the different culling capacity used, up to 20 farms per day in Backer et al. [74] vs only up to six farms per day in Seymour et al. [75]. This difference highlights the importance of this parameter on the evaluation of control strategies. Overall, these results also emphasize the importance of comparing several models with different structures and assumptions to inform policy recommendations [85, 86].

### Model limitations and future avenues for modeling AIV transmission

#### Research gaps on avian influenza transmission and control

It is worth noting that until the winter of 2021–2022, the HPAI H5N8 subtype was responsible for the largest epidemics ever recorded in the EU in terms of the number of poultry outbreaks, geographical extent, and the number of dead wild birds, spreading across more than 29 European countries in the winters of 2016–2017 and of 2020–2021 [87]. However, only three studies in our review focused on parameter estimation and evaluation of control strategies of HPAI H5N8 in the EU [62–64]. Given the animal welfare and economic impact that these epidemics had, further research is needed on mechanistic modeling of HPAI H5N8 to better understand how the virus spread and how best to control it [32]. Since 2021, the HPAI H5N1 subtype has become predominant and caused thousands of outbreaks in domestic poultry [87–89]. Modeling studies will therefore also be required for this new devastating and widespread epidemic. In particular, the evaluation of vaccination in some European countries should be a priority given that it may become part of their strategy to mitigate the impact of HPAI [16].

Only five studies in our review included data on LPAI viruses, including three studies on the LPAI H7N9 viruses that were first discovered in China in 2013. Given their zoonotic potential and the ability of H5 and H7 LPAI viruses to mutate into HPAI, further research is needed on this topic [32]. This is especially true as each virus subtype and context is unique, and therefore a better understanding of the transmission of LPAI viruses in different settings is needed.

#### Fine-tuning models

Models are created for a specific situation with three key (and often opposing) characteristics to be balanced: accuracy (the ability to reproduce the observed data), tractability (the capacity to solve and understand the model analytically or numerically), and flexibility (the ease with which the model can be adapted to new situations) [90]. The trade-off between these characteristics depends on the question being addressed and the availability of data. Even when data are available, simplification is often necessary, for example to get a more flexible and tractable model or save computational time. In doing so, some processes that could impact transmission dynamics may be overlooked.

In the articles reviewed, this was the case for the impact of host species and production systems. Many models (24/46) considered a homogeneous “poultry” population. However, as was demonstrated in some of the articles, galliformes and palmipeds contributed differently to different epidemics [36, 60, 63], and transmission parameters may vary between species depending on the virus subtype [32, 36, 60, 63] and between commercial and backyard farms [68, 72]. Thus, caution should be taken in pooling all poultry populations into one. Whenever possible and according to the available data, models should account for heterogeneity between host species and production systems to gain a better understanding of the contribution of each poultry population to AIV transmission and design control strategies accordingly. Even if host heterogeneity is not included in the model, future studies should at least report information on which poultry populations are included, e.g., to clarify which parameter values are applicable to which species [32].

Another modeling assumption that should be refined is the homogeneous mixing of individual birds, especially at the national [52, 53, 66] and global scales [44, 45]. This assumption means that every host interacts uniformly and randomly with every other host with the same probability, thus neglecting any heterogeneity that may arise from differences in age, behavior, and most importantly, spatial location [90]. This assumption helps simplify models in the absence of detailed data, and may be appropriate at small scales, such as for within-farm epidemic dynamics [35, 57, 62, 64, 65, 73, 74, 76, 77]. However, it may not adequately reflect transmission dynamics at larger scales.

Similarly, the homogeneous mixing of farms [39, 41, 43, 48, 68, 69, 72] may not be adequate, especially for large geographical areas. To refine this assumption and introduce spatial heterogeneity, spatial kernels were most of the time included at the between-farm transmission scale (Figure 3). This may represent a simple but adequate alternative that is more representative of the spatial spread of AIV than homogeneous mixing.

Finally, the scale of the epidemiological unit was also important. Eight articles used administrative areas as the epidemiological unit [36, 37, 40, 49–51, 53, 55]. These administrative areas ranged from villages or communes up to US counties or Nigerian states. In all these articles, the administrative area was considered as a single unit, i.e., the whole area became exposed or infectious at the same time, neglecting within-area dynamics. Rorres et al. [55] compared models using three different epidemiological units in Pennsylvania (US): farms, ZIP-codes and counties. Interestingly, the epidemic dynamics were consistent with those at the farm level when aggregating at the ZIP-code level, but not at the county level. These results suggest that US counties are too large to neglect the within-area transmission dynamics [55]. Similar results were found when producing risk maps in the Netherlands: similar risk maps were obtained at the farm or municipality level, but not at the regional level [31]. Therefore, we recommend, depending on the available data and the modeling objective, to use the smallest spatial resolution possible.

#### Estimating parameters from field data

In most studies, only the transmission parameter was estimated using field data (Table 3). Other parameters, such as the frequently used average durations of the latent and infectious periods, were more often assumed (Additional files 4 and Additional file 5).

For instance, most within-farm model parameters were assumed based on experimental studies for the same or different virus subtype. However, the course of the infection could be different in field situations compared to experimental studies, and could also differ between virus subtypes. Therefore, further insights could be gained on within-farm transmission dynamics and on the course of infection in individual birds by estimating parameters such as the durations of the latent and infectious periods from field outbreak data, when possible. Of course, results from experimental studies could still be used when these parameters cannot be estimated, or as a priori knowledge if the estimation is performed in a Bayesian framework [64]. The introduction time of the virus is another parameter that was estimated in only a few studies, despite its importance for contact tracing and identifying risk factors for the introduction of AIVs in a farm [62]. Using modeling approaches and epidemic data to estimate this parameter thus represents a potential avenue for future research. Moreover, this parameter could be used to inform the time between the onset of infection and its detection (incubation period) in between-farm transmission models.

The duration of the incubation period at the farm level was estimated in only four of the 24 articles that considered between-farm transmission (Additional file 5). Results from within-farm modeling could be used to better inform this parameter and how it varies between farms according to their characteristics (species, size…). Alternatively, this parameter could be estimated, as was done in Kim et al. [42], Hill et al. [46] and Andronico et al. [63]. This would provide useful information on the effectiveness of surveillance.

#### Improving the evaluation of control strategies

Given the frequency of devastating epidemics and the potential switch toward endemic circulation of HPAI viruses worldwide, preparedness and prevention strategies are critical for the sustainability of poultry production. Mechanistic models of AIV transmission are highly valuable since they provide the possibility to compare several strategies in a given epidemiological setting, which is rarely feasible in the field. The evaluation of different combinations of AIV control strategies deserves further attention. Yet except for combining various strategies with the conventional practice of reactive culling, most articles included in this review compared strategies in isolation. However, the repeated occurrence of devastating HPAI H5Nx epidemics around the world raises serious concerns about the capacity of existing strategies to control HPAI viruses, and highlights that no strategy alone is sufficient [18]. For instance, investigating the use of vaccination in combination with other control strategies could provide useful insights. The barriers to vaccine usage in the EU are progressively being removed, especially with the catastrophic impact of the recent epidemics of HPAI H5N1. The Netherlands and France, among others, already have performed clinical trials of vaccines [91, 92]. Mechanistic models can help assess the effectiveness of control strategies, including vaccination. The implementation of additional mitigation approaches, such as reinforcing biosecurity measures and reducing the density of poultry farms, is a prerequisite to successful vaccination campaigns [18]. In this context, mechanistic models offer a promising route to decipher the effects of associated mitigation and control strategies.

Furthermore, greater attention needs to be paid to the economic costs of control strategies. Effective control strategies from an epidemiological point of view do not necessarily translate into efficient strategies from an economic point of view. In this review, only three articles evaluated the economic costs of an epidemic under various control strategy scenarios [38, 74, 75]. Further work combining mechanistic epidemiological models with economic models is therefore needed.

While no strategy can be used in every context, future investigations could be done with optimization algorithms that incorporate the parameters defining the mitigation strategies as input to determine the set of strategies required to attain the desired outcomes (e.g., minimize the overall epidemic or economic impact) [93]. This approach could complement the traditional method of comparing a set of predefined control strategies. Examples of parameters could be pre-emptive culling and vaccination efforts, and this method would find the optimal strategy (i.e., how much culling and how much vaccination is required) to reach the objective (e.g., minimizing the epidemic impact). Moreover, this could also help in designing time-varying control strategies [94]. The optimal control measure may indeed change over time according to epidemic dynamics and the effects of the interventions themselves. Such optimization approaches were used, for example, to determine the cost-effective surveillance strategies for invasive species management in Australia [95] and to choose the optimal allocation of resources between quarantine and surveillance for protecting islands from pest invasion [93].

Real-time modeling could also provide useful insights to decision makers by comparing scenarios in the early stages of an ongoing epidemic [96]. However, limited data and the resulting uncertainty in parameter values may limit the ability of models to provide useful insights. In this review, only one article retrospectively assessed the ability of its model to be used in real-time by evaluating interventions with models fitted to available data at different time points during the epidemic [38]. Interestingly, despite high uncertainty in model projections, the ranking of control measures was consistent early on during the course of the epidemic (even when using only the first 10 days of the epidemic) when the objective was to reduce the epidemic size or the number of culled flocks. Similar results were found for two epidemics of foot-and-mouth disease [96], indicating the ability of models to provide accurate comparisons of interventions relatively early during an epidemic. However, when the objective was to reduce the epidemic duration, using more data (25 days) was needed to obtain consistent rankings. This means that recommendations made to policy makers could be wrong if they were made before a sufficient amount of data was available [38]. Further research is therefore needed to assess the models’ ability to be used in real-time for the evaluation of control strategies.

Finally, we identified in our review the importance of accounting for limited management capacity in mechanistic models of HPAI. Not accounting for limited resources may overestimate the efficacy of interventions [47, 50, 60, 63, 70, 74, 75], which may lead to suboptimal or even wrong recommendations to policy makers. However, it may be difficult to quantify culling or vaccination capacities and how these change over the course of an epidemic. Different assumptions regarding this parameter may lead to different conclusions, as seen for the 2003 HPAI H7N7 epidemic in the Netherlands [74, 75]. How to accurately model limited management capacity, and how this capacity changes over the course of an epidemic, is therefore an avenue for future research. Collaboration with veterinary services and policy makers will be crucial to make realistic assumptions about this parameter, among others.

### Supplementary Information

**Additional file 1:** Complete overview of search terms used in PubMed, Web of Science and CAB Abstracts.

**Additional file 2: **Summary of the two-step screening.

**Additional file 3: **Data extraction from the 46 included articles [35–80].

**Additional file 4: **Parameter values used in within-farm transmission models [35, 57, 62, 64, 65, 73, 74, 76, 77, 100–135].

**Additional file 5: **Parameter values used in between-farms transmission models. [38, 41–43, 46–48, 50, 54–56, 59–61, 63, 67–72, 75, 101, 102, 116, 122, 126, 129, 136–142].

## Data Availability

All data generated or analyzed during this study are included in this published article and its Additional files.
