# Supplementary material for: A systematic review of mechanistic models used to study avian influenza virus transmission and control
Source: Vet Res. 2023 Oct 18;54:96. doi: 10.1186/s13567-023-01219-0 (PMC10585835; doi:10.1186/s13567-023-01219-0)
Supplement: Supplementary file 1 — Additional file 1: Complete overview of search terms used in PubMed, Web of Science and CAB Abstracts. [file 13567_2023_1219_MOESM1_ESM.docx]

**Additional file 1:** **Complete overview of search terms used in PubMed, Web of Science and CAB Abstracts.**

| **Database** | **Search terms** | **Hits*** |
| --- | --- | --- |
| PubMed | ((("avian influenza"[Title/Abstract] OR "bird flu"[Title/Abstract])  AND  (model*[Title/Abstract] OR simulat*[Title/Abstract] OR quantitative[Title/Abstract]))  AND  (dynamic*[Title/Abstract] OR determinist*[Title/Abstract] OR stochas*[Title/Abstract] OR compartmen*[Title/Abstract] OR transmission[Title/Abstract] OR mechanist*[Title/Abstract] OR quantitative[Title/Abstract] OR metapopulation[Title/Abstract])) | 967 |
| Web of Science | TS=(“avian influenza” OR “bird flu”)  AND  TS=(model* OR simulat* OR quantitative)  AND  TS=(dynamic* OR determinist* OR stochas* OR compartmen* OR transmission OR mechanist* OR quantitative OR metapopulation)  **Refined by:** **DOCUMENT TYPES:** (ARTICLE ) | 938 |
| CAB Abstracts | (title:("avian influenza" OR "bird flu") OR ab:("avian influenza" OR "bird flu") OR subject:("avian influenza" OR "bird flu"))  AND  (title:(model* OR simulat*) OR ab:(model* OR simulat* ) OR subject:(model* OR simulat* ))  AND  (title:(dynamic* OR determinist* OR stochas* OR compartmen* OR transmi* OR mechanistic* OR quantit* OR metapop*) OR ab:(dynamic* OR determinist* OR stochas* OR compartmen* OR transmi* OR mechanistic* OR quantit* OR metapop*) OR subject:(dynamic* OR determinist* OR stochas* OR compartmen* OR transmi* OR mechanistic* OR quantit* OR metapop*) | 1015 |

* Number of hits before deduplication.
