# Supplementary material for: A systematic review of mechanistic models used to study avian influenza virus transmission and control
Source: Vet Res. 2023 Oct 18;54:96. doi: 10.1186/s13567-023-01219-0 (PMC10585835; doi:10.1186/s13567-023-01219-0)
Supplement: Supplementary file 2 — Additional file 2: Summary of the two-step screening. [file 13567_2023_1219_MOESM2_ESM.docx]

**Additional file 2: Summary of the two-step screening.**

| **First step screening (title and abstract level)** | |
| --- | --- |
| Publication type | Does the study involve a primary research article on avian influenza?   - Yes (include) - No (exclude)^1^ |
| Population | Does this article study avian influenza in poultry?   - Yes (include) - No (exclude)^2^ |
| Study type | Does this article study the propagation of the influenza epidemic at the population level?   - Yes (include) - No (exclude)^3^ |
| Model type | Does this article describe a mechanistic approach in studying an avian influenza outbreak?   - Yes (include) - No (exclude)^4^ |
| **Second step screening (full text)** | |
| Publication type | Does the study involve a primary research article on avian influenza?   - Yes (include) - No (exclude)^1^ |
| Population | Does this article study avian influenza in poultry?   - Yes (include) - No (exclude)^2^ |
| Study type | Does this article study the propagation of the influenza epidemic at the population level?   - Yes (include) - No (exclude)^3^ |
| Model type | Does this article describe a mechanistic approach in studying an avian influenza outbreak?   - Yes (include) - No (exclude)^4^ |
| Model objective | Does this article present a model that was used to estimate transmission parameters using avian influenza epidemic data and/or to evaluate mitigation strategies after being fitted to avian influenza epidemic data?   - Yes (include) - No (exclude)^5^ |

^1^ Articles to be excluded are reviews, commentaries, editorials, and perspective papers.

^2^ Articles to be excluded are those that do not focus on avian influenza in at least one domestic poultry species.

^3^ Articles to be excluded are those that focus on experimental or molecular studies.

^4^ Articles to be excluded are those using a non-mechanistic approach. By mechanistic approach, we mean compartmental or individual-based (non-exhaustive list) models where the process of infection was made explicit.

^5^ Articles to be excluded are those using theoretical models that have not been fitted on avian influenza epidemics (HP or LP) data.
