# Supplementary material for: A systematic review of mechanistic models used to study avian influenza virus transmission and control
Source: Vet Res. 2023 Oct 18;54:96. doi: 10.1186/s13567-023-01219-0 (PMC10585835; doi:10.1186/s13567-023-01219-0)
Supplement: Supplementary file 4 — Additional file 4: Parameter values used in within-farm transmission models [35, 57, 62, 64, 65, 73, 74, 76, 77, 100–135]. [file 13567_2023_1219_MOESM4_ESM.docx]

**Additional file 4: Parameter values used in within-farm transmission models.**

| **Reference** | **Subtype** | **Average duration of the latent period (days)** | | **Average duration of the infectious period (days)** | | **Transmission rate** $\boldsymbol{\beta}$ **(day^-1^)** | | **Case fatality risk (%)** | | **Reproduction number** $\boldsymbol{R}_{\boldsymbol{0}}$ | |
| --- | --- | --- | --- | --- | --- | --- | --- | --- | --- | --- | --- |
|  |  | **Value** | **Sources** | **Value** | **Sources** | **Value** | **Sources** | **Value** | **Sources** | **Value** | **Sources** |
| Tiensin et al. [35] | HP/H5N1 | - | - | Min: 1  Max: 4 | [100–103] | Min: 0.60 (0.43-0.84)  Max: 2.30 (1.92-2.76) | Estimated | 100 | [104, 105] | Min: 2.18 (1.94-2.46)  Max: 3.49 (2.70-4.50) | Estimated |
| Ssematimba et al. [57] | HP/H5N2 | 1 | [106, 107] | 4 | [106, 107] | 3.2 (2.3-4.3) | Estimated | 100 | [106–111] | 12.8 (9.2-17.2) | Estimated |
| Hobbelen et al. [62] | HP/H5N8 | Min: 1  Max: 2 | [112–114] | Min: 1.1  Max: 8.5 | [112–124] | Min: 0.95 (0.3-2.3)  Max: 34.4 (27.3-44.1) | Estimated | Min: 20  Max: 100 | [112–124] | Min: 5.225 (1.65-12.65)  Max: 86 (68.25-110.25) | Estimated |
| Vergne et al. [64] | HP/H5N8 | 0.17 (0.03-0.38) | Estimated | 4.3 (2.8-5.7) | Estimated | 4.1 (2.8-5.8) | Estimated | 70 (61-78) | Estimated | 17.5 (9.4-29.3) | Estimated |
| Hayama et al. [65] | HP/H5N8 | 2 | [125] | 4 | [125] | Min: 0.661 (0.627-0.696)  Max: 3.387 (1.774-2.071) | Estimated | 100 | Assumed | Min: 2.642 (2.507-2.785)  Max: 13.548 (12.832-14.304) | Estimated |
| Bos et al. [73] | HP/H7N7 | - | - | Min: 4  Max: 15 | [126] | 4.50 (2.68-7.57) | Estimated | 70 | [126] | - | - |
| Backer et al. [74] | HP/H7N7 | 1 | [126–128] | 4 | [126, 127, 129] | 1.9 (0.61-8.1) | Estimated | 70 | [126, 127] | 7.6 (2.44-32.4) | Estimated |
| Bonney et al. [76] | LP/H5N2 | 0.63 | [130–132] | 11.78 | [130–135] | Min: 0.6 (0.4-1.0)  Max: 3.9 (1.2-5.8) | Estimated | 1 | Assumed | Min: 7.068 (4.712-11.78)  Max: 45.942 (14.136-68.324) | Estimated |
| Gonzales et al. [77] | LP/H7N3 | - | - | Min: 7.69 (5.88-11.11)  Max: 9.09 (6.25-20.0) | Estimated | Min: 0.50 (0.45-0.55)  Max: 0.72 (0.68-0.77) | Estimated | 0 | Assumed | Min: 4.7 (3.0-8.6)  Max: 5.6 (4.3-7.7) | Estimated |

When several values were reported, minimum and maximum values are indicated. For estimated parameter values, the mean and 95% confidence interval (or median and 95% credible interval) are indicated. The unit of the transmission parameter $\beta$ was always day^-1^, as all models assumed frequency-dependent contact rates and homogeneous mixing between birds within a flock.
