# Supplementary material for: A systematic review of mechanistic models used to study avian influenza virus transmission and control
Source: Vet Res. 2023 Oct 18;54:96. doi: 10.1186/s13567-023-01219-0 (PMC10585835; doi:10.1186/s13567-023-01219-0)
Supplement: Supplementary file 5 — Additional file 5: Parameter values used in between-farms transmission models. [38, 41–43, 46–48, 50, 54–56, 59–61, 63, 67–72, 75, 101, 102, 116, 122, 126, 129, 136–142]. [file 13567_2023_1219_MOESM5_ESM.docx]

**Additional file 5: Parameter values used in between-farms transmission models.**

| **Reference** | **Subtype** | **Value** | **Sources** |
| --- | --- | --- | --- |
| **Average duration of the latent period (from onset of infection to onset of infectiousness) in days** | | | |
| Pelletier et al. [50] | HP/H5N1 | 5 | Assumed |
| Rorres et al. [54] | HP/H5N2 | 4 | Estimated |
| Rorres et al. [55] | HP/H5N2 | 5 | [136] |
| Bonney et al. [56] | HP/H5N2 | 3 | Assumed |
| Salvador et al. [59] | HP/H5N6 | Min: 1 – Max: 2 | [67, 101, 126, 137] |
| Yoo et al. [61] | HP/H5N6 | 1 | Assumed |
| Andronico et al. [63] | HP/H5N8 | 1 | Estimated |
| Dorigatti et al. [67] | HP/H7N1 | 2 | [129] |
| Smith and Dunipace [68] | HP/H7N3 | 2 | [71] |
| Stegeman et al. [69]  Le Menach et al. [70]  Boender et al. [71]  Bavinck et al. [72] | HP/H7N7 | 2 | [129, 138, 139] |
| **Average duration of the infectious period (from onset of infectiousness to culling) in days** | | | |
| Delabouglise et al. [41] | HP/H5N1 | Min: 4 – Max: 13 | Assumed |
| Ssematimba et al. [48] | HP/H5N1 | Min: 7.9 – Max: 12 | Estimated |
| Pelletier et al. [50] | HP/H5N1 | 10 | Assumed |
| Rorres et al. [54] | HP/H5N2 | 7 | Assumed |
| Rorres et al. [55] | HP/H5N2 | 7 | [136] |
| Bonney et al. [56] | HP/H5N2 | 17.22 (16.57-17.87) | Estimated |
| Dorigatti et al. [67] | HP/H7N1 | 11.82 (6-26) | Estimated |
| Stegeman et al. [69] | HP/H7N7 | Min: 6.9 (3.9-9.9)  Max: 13.8 (9.9-17.6) | Estimated |
| Le Menach et al. [70] | HP/H7N7 | Min: 6 – Max: 10 | Assumed |
| Boender et al. [71] | HP/H7N7 | 7.47 (7.2-7.8) | Estimated |
| Seymour et al. [75] | HP/H7N7 | 6.4 | Estimated |
| **Average duration of the incubation period (from onset of infection to reporting) in days** | | | |
| Delabouglise et al. [41] | HP/H5N1 | Min: 3 – Max: 12 | Assumed |
| Kim et al. [42] | HP/H5N1 | Min: 2.5 – Max: 7.2 | Estimated |
| Kim and Cho [43] | HP/H5N1  HP/H5N8  HP/H5N6 | Min: 2 – Max: 8 | [102, 116, 122, 140, 141] |
| Hill et al. [46, 47] | HP/H5N1 | 7 | Estimated |
| Ssematimba et al. [48] | HP/H5N1 | 7 | Assumed |
| Bonney et al. [56] | HP/H5N2 | 8 | Assumed |
| Salvador et al. [59] | HP/H5N6 | Min: 10 – Max: 14 | Assumed |
| Lee et al. [60] | HP/H5N6  HP/H5N8 | Min: 2 – Max: 4 | Assumed |
| Yoo et al. [61] | HP/H5N6 | Min: 5.87 (1-12.85)  Max: 5.98 (1-12.94) | Estimated |
| Andronico et al. [63] | HP/H5N8 | 7 | Estimated |
| Dorigatti et al. [67] | HP/H7N1 | 7 | [142] |
| Smith and Dunipace [68] | HP/H7N3 | Min: 3 – Max: 9 | Assumed |
| Stegeman et al. [69]  Le Menach et al. [70]  Boender et al. [71] | HP/H7N7 | 6 | Assumed |
| Bavinck et al. [72] | HP/H7N7 | Min: 4 – Max: 12 | Assumed |

(continued)

| **Reference** | **Subtype** | **Value** | **Sources** |
| --- | --- | --- | --- |
| **Average duration of the clinical period (from reporting to culling) in days** | | | |
| Delabouglise et al. [41] | HP/H5N1 | 1 | Assumed |
| Salvador et al. [59] | HP/H5N6 | Min: 1 – Max: 3 | Assumed |
| Yoo et al. [61] | HP/H5N6 | 0 | Assumed |
| Andronico et al. [63] | HP/H5N8 | 5.2 | Estimated |
| Smith and Dunipace [68] | HP/H7N3 | Min: 0 – Max: 9.56 | Estimated |
| Le Menach et al. [70] | HP/H7N7 | Min: 1 – Max: 6 | [129] |
| **Transmission rate** | | | |
| Retkute et al. [38] | HP/H5N1 | 0.99 (0.76-1.12) ×10^-6^ | Estimated |
| Delabouglise et al. [41] | HP/H5N1 | Min: 1.4 ×10^-8^ – Max: 40 ×10^-8^ | Estimated |
| Kim et al. [42] | HP/H5N1 | 0.429 (probability) | Estimated |
| Hill et al. [46, 47] | HP/H5N1 | Min: 1.71 (0.586-3.63) ×10^-10^  Max: 1.06 (0.0729-3.78) ×10^-7^ | Estimated |
| Ssematimba et al. [48] | HP/H5N1 | Min: 0.08 (0.06-0.10)  Max: 0.11 (0.08-0.20) | Estimated |
| Bonney et al. [56] | HP/H5N2 | 0.0061 (0.0025-0.0137) | Estimated |
| Salvador et al. [59] | HP/H5N6 | 0.0012 (0.0001-0.1) | Estimated |
| Lee et al. [60] | HP/H5N6  HP/H5N8 | Min: 0.00007 – Max: 0.00707 | Estimated |
| Andronico et al. [63] | HP/H5N8 | Min: 0.23 (0.16-0.31)  Max: 0.53 (0.37-0.72) | Estimated |
| Dorigatti et al. [67] | HP/H7N1 | Min: 0.0009 (0.0005-0.0013)  Max: 0.0155 (0.0078-0.0232) | Estimated |
| Smith and Dunipace [68] | HP/H7N3 | Min: 0 – Max: 0.00238 | Estimated |
| Stegeman et al. [69] | HP/H7N7 | Min: 0.17 (0.1-0.2)  Max: 0.47 (0.3-0.7) | Estimated |
| Le Menach et al. [70] | HP/H7N7 | Min: 0.076 – Max: 0.336 | Estimated |
| Boender et al. [71] | HP/H7N7 | 0.002 (0.0012-0.0039) | Estimated |
| Bavinck et al. [72] | HP/H7N7 | 1.7 (1.5-2.0) ×10^-4^ | Estimated |
| Backer et al. [74] | HP/H7N7 | 0.0039 (0.0023-0.0076) | Estimated |
| **Reproduction number** $\boldsymbol{R}_{\boldsymbol{h}}$ | | | |
| Delabouglise et al. [41] | HP/H5N1 | Min: 0.55 – Max: 15.7 | Estimated |
| Kim and Cho [43] | HP/H5N1  HP/H5N8  HP/H5N6 | Min: 0.03 (0-0.98)  Max: 2.20 (1.51-3.16) | Estimated |
| Ssematimba et al. [48] | HP/H5N1 | Min: 0.85 (0.77-1.02)  Max: 0.96 (0.72-1.20) | Estimated |
| Smith and Dunipace [68] | HP/H7N3 | 4.8 | Estimated |
| Stegeman et al. [69] | HP/H7N7 | Min: 1.2 (0.6-1.9)  Max: 6.5 (3.1-9.9) | Estimated |
| Bavinck et al. [72] | HP/H7N7 | 1.33 | Estimated |

When several values were used, minimum and maximum values are indicated. For estimated parameter values, the mean/median and 95% confidence/credible interval (when reported) are indicated.
